# Association of the rs2111234, rs3135499, rs8057341 polymorphisms in the NOD2 gene with leprosy: A case-control study in the Norte de Santander, Colombia population

**DOI:** 10.1371/journal.pone.0281553

**Published:** 2023-03-06

**Authors:** Mónica Alexandra Bustos, Luz Dary Castañeda-Castañeda, Carmen Rosa Acosta, Diana García, Diana Patricia Bohada, Raúl Rodríguez, Martha Inírida Guerrero

**Affiliations:** 1 Grupo de Investigación en Enfermedades Parasitarias, Tropicales e Infecciosas (GIEPATI), Universidad de Pamplona, Pamplona, Norte de Santander, Colombia; 2 Grupo de Dermatología General, Hospital Universitario- Centro Dermatológico Federico Lleras Acosta, Bogotá, Colombia; 3 Research Institute, Group of Basic Sciences in Health (CBS)-FUCS, Fundación Universitaria de Ciencias de la Salud-FUCS, Bogotá, Colombia; 4 Grupo de Dermatología Tropical, Hospital Universitario- Centro Dermatológico Federico Lleras Acosta, Bogotá, Colombia; International Medical University, MALAYSIA

## Abstract

**Background:**

Leprosy is a chronic infectious disease caused by *Mycobacterium leprae*. The development of leprosy involves several factors, including the causative agent, the individual host’s immune response, environmental factors, and the genetic background of the host. Specifically, the host’s innate immune response, encoded by genes, determines their susceptibility to developing leprosy post-infection. Polymorphic variants in the nucleotide-binding oligomerization domain 2 (*NOD2)* gene are associated with leprosy among populations in a variety of endemic areas around the world. Colombia, a country located in the tropical zone, has several leprosy-endemic regions, including Norte de Santander. The aim of this study was to analyze the rs7194886, rs2111234, rs3135499, and rs8057341 single nucleotide polymorphisms (SNPs) in the NOD2 gene using a case-control study to determine whether they confer greater or lesser susceptibility to the development of leprosy.

**Methodology:**

The TaqMan qPCR amplification system was used for SNPs detection.

**Findings:**

An association between the A-rs8057341 SNP (p = 0,006286) and resistance to leprosy was found. However, the rs3135499 (*p = 0*,*9063*) and rs2111234 (*p = 0*.*1492*) were not found to be associated with leprosy susceptibility. In addition, the rs7194886 SNP was not found to be in Hardy-Weinberg equilibrium (HWE) in the study population. The GAG haplotype, consisting of SNPs rs2111234-G, rs3135499-A, and rs8057341G, acts as a susceptibility factor for the development of leprosy in women. SNPs rs3135499 and rs8057341 are functionally related to decreased NOD2 expression according to an *in-silico* analysis.

**Conclusions:**

The SNPs rs8057341-A was related with resistance to leprosy and the haplotype rs2111234-G, rs3135499-A and rs8057341-G SNPs was related with susceptibility in the Norte de Santander Colombia, studied population.

## Introduction

Leprosy is a chronic granulomatous disease [1, 2], characterized by mainly affecting skin macrophages and Schwann cells in the peripheral nerves in humans. The etiologic agent is *Mycobacterium leprae (M*. *leprae*), an acid-fast, obligate intracellular bacillus, that is refractory to in vitro culture but is successfully cultivated in armadillo, chimpanzee and mouse animal models [3]. The clinical manifestations of the disease are varied, however, the genetic variability of the bacillus is low [4, 5], providing evidence on the importance of individual host immune response, microenvironment and genetic characteristics, in the development of this disease [6].

During infection, *M*. *leprae* antigens interact with extracellular and intracellular pattern recognition receptors (PRRs) in the host cell. Nucleotide-binding oligomerization domain (NOD)-like receptors (NLRs) are a group of intracellular PRRs. Single-nucleotide polymorphisms (SNPs) in genes encoding these proteins may contribute to susceptibility to infectious diseases such as leprosy [7]. NLRs encompass the *NOD2* gene or *CARD15* gene, located on chromosome 16q12-21 which encodes the nucleotide-binding oligomerization domain-containing protein 2(NOD2) [8]. Protein NOD2 consists of 1040 amino acids featuring three regions: a carboxy-terminal domain rich in leucin repeats (LRR), which is involved in antigen recognition, a central nucleotide-binding domain (NBD) that facilitates self-oligomerization and contains the ATP-binding site and a carboxy-terminal domain composed of an N-terminal caspase recruitment domain (CARD) or Pyrin domain [9], which is responsible for signal transduction through protein-protein interactions. NOD2 possess two tandem repeats of CARD regions activated by muramyl dipeptide (MDP), which is a component of the cell wall [10]. The *Mycobacterium leprae* MDP is structurally distinct compared to other mycobacteria, however, it maintains its ability to induce NOD2 signaling pathway activation [11].

The recognition of MDP by NOD2 triggers modifications in protein conformation, allowing unfolding followed by oligomerization and exposure of the CARD domain. ATP -binding is required for NBD-mediated protein oligomerization to occur. Followed by serine/threonine protein kinase 2 (RIP2 or RICK) recruitment. The RICK -NOD2 complex is required to induce NEMO protein (IKK -γ; inhibitor of nuclear factor Kappa- B kinase- gamma kinase) polyubiquitination and interaction and triggers NF-*k*B activation [12]. MDP-induced NOD2 stimulation regulates numerous biological processes, including autophagy [12, 13].

Several studies have reported that polymorphic variation in the NOD2 gene affects susceptibility to leprosy development, though the nature of the association is population-dependent. The rs12448797, rs2287195, rs8044354, and rs1477176 variants are associated with susceptibility to leprosy in a population in Nepal [14]. In the same study, the rs7194886 variant was associated with reversal reactions in leprosy, while the rs3135499 variant did not impact disease development in this population [14]. The rs9302752 and rs7194886 variants are associated with the development of leprosy in China [15]. In Vietnam, the rs9302752 SNP confers susceptibility to developing leprosy [14], while the rs7194886 variant does not [16]. A Yi-China population-based study on SNPs s9302752, rs7194886, rs8057341, and rs3135499 found that only rs3135499 associated with the development of leprosy [17]. Furthermore, studies conducted in Brazil showed that SNPs rs8057341-A and rs2111234-G are protective against leprosy development [18]. Taken together, these reports illustrate the variability in the association between gene SNPs and leprosy susceptibility according to the specific leprosy endemic regions [19]. The Norte de Santander region has the fourth highest incidence of leprosy in Colombia [20]. Therefore, this study aimed to identify any associations between the rs3135499, rs2111234, rs8057341, and rs7194886 SNPs in the *NOD2* gene and susceptibility to leprosy in a sample of leprosy patients and healthy individuals from this region in northern Colombia. In addition, analyses stratified by age and sex were performed to further characterize the relationship between these polymorphisms and leprosy.

## Materials and methods

### Ethics declaration

This study was evaluated and reviewed for ethical aspects and approved by the ethics committee of the Federico Lleras Acosta University Hospital Dermatology Center in Bogotá, Colombia (MinCiencias:212084368694- Code:1DIS02-2Ñ). The ethical principles for medical research involving human subjects outlined in the Helsinki Declaration were followed. This study was considered to have minimal risk. All legal-age participants (above 18 years old) provided written informed consent to participate in the present study. The inclusion of patients under the legal age required the written informed consent of their parents or legal guardian. The control subjects agreed to participate by signing a different informed consent document. The STREGA guidelines were followed throughout this study (S1 Table).

### Study subjects

This case-control study was conducted in Norte de Santander, Colombia, from 2020 to 2021. We calculated the sample size needed to produce an odds ratio [OR] > 1.8 with 80% power, α = 0. 05, and 0.2 type II error. SNPs with allelic frequencies greater than 0.10, according to a dbSNP reference database, were chosen for analysis. We included 570 individuals distributed among cases (n = 114) and controls (n = 456) at a control-to-case ratio of 4:1. All of the included individuals were born and resided in Norte de Santander.

The Public Health System records (SIVIGILA) of the leprosy program in Norte de Santander were reviewed, and patients older than 18 years of age or minors with a confirmed diagnosis of leprosy were selected. Only patients with complete, verifiable data in the program´s database who were still alive were included.

The control group included healthy subjects who were born and resided in Norte de Santander without a diagnosis of leprosy or link to it, older than 18 years of age, and with no suspicion or diagnosis of an autoimmune disease. People with a family history of leprosy among first and second-degree relatives or who lived in the same household with a patient diagnosed with leprosy were excluded.

### Polymorphism selection

Genome-wide association studies (GWAS), family-based studies, and case-control studies performed in various countries around the world, including China [15], Vietnam [16], Brazil [18, 21], and India [22] among others, informed our genotyping strategy in the Norte de Santander population. We chose four polymorphisms (SNPs) in the *NOD2*, gene: rs3135499, rs2111234, rs8057341, and rs7194886. Each variant was evaluated in the online Regulome DB database to predict its functional significance (https://www.regulomedb.org/regulome-search/).

### Data collection

Data was collected for sociodemographic characteristics from the SIVIGILA program databases, which include the address and telephone number of both the patient and a family member; potential participants were contacted and recruited in the different municipalities of Norte de Santander. Control group participants were recruited from primary care practices in different places such as universities, health centers and other entities of said municipalities. The S2 Table was used for data collection.

### Sample collection

A 4 ml blood sample was collected in tubes containing ethylene diamine tetraacetic acid (EDTA) anticoagulant. Samples were stored at room temperature and transported to the processing laboratory within three hours. The samples were centrifuged at 2500 rpm for 10 minutes to separate the leukocytes, from which DNA extraction was performed. PureLink Genomic DNA extraction Kits (Invitrogen ^®^), were used following the manufacturer´s instructions. The extracted DNA was subjected to quantification using a NanoDrop™ ND-1000 (ThermoFisher^®^, USA) spectrophotometer, and subsequently diluted and stored at -20°C until genotyping.

### Genotyping

The rs3135499 (assay ID c_31758802_10), rs2111234 (assay ID c_15820716_10), rs8057341 (assay ID c_3017466_10) and rs7194886 (C_3017470_10) variants were identified with pre-designed and validated TaqMan^TM^ probe genotyping assays obtained from Life Technologies (Invitrogen^®^) in the StepOnePlus™ Real-Time PCR System kit. Allelic discrimination was conducted following the manufacturer´s instructions. The Real-Time PCR reaction was performed in a final volume of 10 µL. The DNA concentration used was 2.5 ng/μl. 0.25 µl of the probe and 5 µL of TaqMan™ Universal PCR Master Mix (Applied Biosystems^®^) were used. Amplification conditions were: 1 cycle at 95°C for 10 m and 40 cycles at 95°C for 15 s and at 60°C for 1 m. A quality control analysis was conducted independently by two investigators to verify the allelic discrimination reported by the assay. In case of non-concordance, the sample was amplified or sequenced again. In addition, 5 samples were amplified by end-point PCR and used for sequencing for each SNP.

### PCR amplification

A DNA segment containing each SNP was amplified from 5 randomly chosen samples to be used as a template for sequencing in order to confirm the allelic discrimination performed with Real time PCR. Primers were designed using primer3 tool (https://primer3.ut.ee/) using the Ensemble (ENST00000647318.2 ENSG00000167207) NOD2 gene annotation system. The primers were evaluated with the *in-silico* PCR UCSC Genome Browser tool (https://genome.ucsc.edu/) to determine their size and accuracy. The PCR sequences and the size of the amplified primer are described in S3 Table. Samples were prepared to a final volume of 20 μl containing 10 μl of OneTaq^®^ 2X Master Mix (NEW ENGLAND BioLabs Inc), 0,4 μl of each primer (10 μM) and 2 μl of DNA (2,5 ng/ μl) and 7,2 μl of MilliQ water. Amplification conditions were initial denaturation at 95°C for 3m and 40 cycles of denaturation at 95°C for 10s, annealing at 60°C for 30 s and extension 72°C for 30s, with a final extension at 72°C for 2 m. PCR products, used for sequencing, were purified using the Monarch^®^ gel extraction kit from New England BioLabs Inc.

### DNA sequencing

The purified products were used as a template for the results for the forward and reverse segment sequences by the Sanger Method. Big Dye Terminator v.3.1 cycle sequencing kit (4336917) Applied Byosystems, Austin, TX, USA was used for the sequencing reaction. Applied Biosystems^®^ GA3500 equipment was used. Sequences were analyzed using free BioEdit v7.2 (Tom Hall; Ibis Biosciences, Carlsbad, CA, USA) and verified in the UCSC Genome Browser (https://genome.ucsc.edu/).

### Statistical analysis

The SNPStats^®^ tool (available at https://www.snpstats.net/start.htm) was used for statistical analyses. Allele and genotypic frequencies were estimated by the direct count method. Hardy Weinberg (H-W) equilibrium was performed using the *X*^2^ test and the 95% confidence interval (CI) for each group (cases vs controls) to assess the distribution bias of genotypes. The SNPStats^®^ program was used to calculate OR by 5-subject-level gene models (dominant, codominant, recessive, over-dominant, and additive). Haplotype frequencies were determined by the expectation-maximization (EM) algorithm using the SNPStats^®^ tool [23]. To estimate haplotype frequencies and association. Linkage disequilibrium (LD) was calculated for four SNPs in the NOD2 gene using the HaploView tool (version 4.2) [24]. Covariables were adjusted for age and sex. *P*-values and statistical significance were defined as *p < 0*.*05*.

## Results

### Demographic characteristics

The mean age of patients with leprosy was 57 (13–86) years and 37 (18–86) years for controls. Gender distribution was similar between the two groups. The female to male ratio in the experimental group was 56/58. Women comprised 51% and 50% of the experimental and control groups, respectively.

### Allele frequency and genotype distribution

Table 1 contains detailed data on the analyzed SNPs, including the allele frequency distribution for each SNP. The rs7194886 SNP was in H-W equilibrium (*p = 0*.*096*) but was not in H-W equilibrium in controls (p = 0.00088); therefore, this SNP was excluded from further analyses. The minor allele was used as the risk allele for the leprosy susceptibility analysis. The A allele of SNP rs8057341 was associated with resistance to leprosy (OR 0.63, 95% CI 0.4681–0.8583, *p = 0*.*0032)*.

**Table 1:** Allele frequencies of 4 NOD2 SNP in 114 leprosy patients and 456 healthy controls from Norte de Santander, Colombia.

SNP ID	Position (GRCh38)	Role	Alleles A/B	MAF		H-W	OR (95% CI)	*p-value* *
				Cases	Controls	*p-value* **		
rs3135499	16:50732216	3’-UTR	A/C	0.36	0.37	0.085	1.06 (0.7873–1.4363)	*0*,*6866*
rs2111234	16:50700122	Intron	G/A	0.38	0.34	0.13	0.77 (0.5762–1.0446)	*0*,*094*
rs8057341	16:50704069	Intron	A/G	0.34	0.45	0.57	0.63 (0.4681–0.8583)	***0*.*0032*** *
rs7194886	16:50691282	2.3kb 5’ NOD2	C/T	0.22	0.24	0.00088 **	0.87 (0.6199–1.2443)	0.4653

SNP, single nucleotide polymorphism; MAF, minor allele frequency; H-W, Hardy Weinberg equilibrium; OR, Odds ratio, CI, Confidence interval

**SNPs with H-W equilibrium, *p < 0*,*05*, were excluded from this analysis

* p-values were calculated using the Chi-square test

The genotype frequencies of the rs3135499, rs2111234, rs8057341, and rs7194886 SNPs in the NOD2 gene in the studied population are shown in Table 2. The genotypic frequencies of SNPs rs3135499 (*p = 0*.*55* and *p = 0*.*085*), rs2111234 (*p = 0*.*84* and *p = 0*. *13*), and rs8057341 (*p = 0*.*53* and *p = 0*.*57*) were found to be in H-W equilibrium in cases and controls respectively. A significant difference in the frequency of rs8057341 genotypes between cases and control group (*p = 0*.*006286)* was found (Table 2).

**Table 2:** Genotypic frequencies of rs3135499, rs2111234, rs8057341, and rs7194886 SNPs in cases and controls (n = 570).

SNP	Genotype	Total n = 570	Controls n = 456 n (frequencies)	Casos	X2	*p-value*
rs3135499	A/A	222 (0.39)	179 (0.39)	43 (0.38)	0,19	0,9063
	A/C	284 (0.5)	227 (0.5)	57 (0.5)		
	C/C	64 (0.11)	50 (0.11)	14 (0.12)		
rs8057341	A/A	104 (0.18)	89 (0.2)	15 (0.13)	10.1388	0.006286**
	G/A	281 (0.49)	233 (0.51)	48 (0.42)		
	G/G	185 (0.32)	134 (0.29)	51 (0.45)		
rs7194886	C/C	315 (0.55)	240 (0.55)	66 (0.58)	1.06893	0.586
	C/T	239 (0.42)	193 (0.42	46 (0.4)		
	T/T	16 (0.03)	14 (0.03)	2 (0.02)		
rs2111234	A/A	177 (0.31)	133 (0.29)	44 (0.39)	3.80542	0.1492
	A/G	295 (0.52)	242 (0.53)	53 (0.46)		
	G/G	98 (0.17)	81 (0.18)	17 (0.15)		

p-value < 0.05

*Statistically significant

### Association between SNPs and susceptibility to leprosy

The different SNPs were evaluated using codominant, dominant, recessive, over-dominant, and additive models. SNP rs8057341 was revealed to be a resistance factor for leprosy development in the codominant model with genotype A/A (OR 0.45; 95% CI 0.22–0.91, *p = 0*.*044*), in the dominant model for genotypes A/G-A/A (OR 0.57; 95% CI 0.36–0.92, *p = 0*.*021*), and the additive model (OR 0.66; 95% CI 0.47–0.92, *p = 0*.*013*) when adjusted for age and gender. The rs2111234 and rs3135499 SNPs were not associated with leprosy onset in any of the analyses, as shown in Table 3.

**Table 3:** Association between single nucleotide polymorphisms (SNPs) and the susceptibility to leprosy according to different inheritance models (n = 570).

SNP_ID	Model	Genotype	Frequencies		Not adjusted		Adjusted	
			Cases	Controls	OR (95% CI)	p-value*	OR (95% CI)	p.-value**
rs8057341	Codominant	G/G	51 (44.7%)	134 (29.4%)	1	**0.0073** ***	1	**0,044** ***
		A/G	48 (42.1%)	233 (51.1%)	**0.54 (0.35–0.85)**		0.62 (0.38–1.03)	
		A/A	15 (13.2%)	89 (19.5%)	**0.44 (0.23–0.84)**		**0.45 (0.22–0.91)**	
	Dominant	G/G	51 (44.7%)	134 (29.4%)	1	**0.0021** ***	1	**0,021** ***
		A/G-A/A	63 (55.3%)	322 (70.6%)	**0.51 (0.34–0.78)**		**0.57 (0.36–0.92)**	
	Recessive	G/G-A/G	99 (86.8%)	367 (80.5%)	1	0.1	1	0,095
		A/A	15 (13.2%)	89 (19.5%)	0.62 (0.35–1.13)		0.58 (0.30–1.12)	
	Over-dominant	G/G-A/A	66 (57.9%)	223 (48.9%)	1	0.085	1	0,32
		A/G	48 (42.1%)	233 (51.1%)	0.70 (0.46–1.05)		0.79 (0.50–1.25)	
	Log-additive	---			**0.63 (0.46–0.86)**	**0.0027** ***	**0.66 (0.47–0.92)**	**0,013** ***
rs3135499	Codominant	A/A	43 (37.7%)	179 (39.2%)	1	0.91	1	0,94
		A/C	57 (50%)	227 (49.8%)	1.05 (0.67–1.63)		0.95 (0.58–1.56)	
		C/C	14 (12.3%)	50 (11%)	1.17 (0.59–2.30)		1.07 (0.51–2.27)	
	Dominant	A/A	43 (37.7%)	179 (39.2%)	1	0.76	1	0,91
		A/C-C/C	71 (62.3%)	277 (60.8%)	1.07 (0.70–1.63)		0.97 (0.61–1.56)	
	Recessive	A/A-A/C	100 (87.7%)	406 (89%)	1	0.69	1	0,78
		C/C	14 (12.3%)	50 (11%)	1.14 (0.60–2.14)		1.11 (0.55–2.21)	
	Over-dominant	A/A-C/C	57 (50%)	229 (50.2%)	1	0.97	1	0,77
		A/C	57 (50%)	227 (49.8%)	1.01 (0.67–1.52)		0.93 (0.59–1.48)	
	Log-additive	---	---	---	1.07 (0.78–1.46)	0.68	1.01 (0.71–1.43)	0,95
rs2111234	Codominant	A/A	44 (38.6%)	133 (29.2%)	1	0.16	1	0,27
		A/G	53 (46.5%)	242 (53.1%)	0.66 (0.42–1.04)		0.75 (0.45–1.24)	
		G/G	17 (14.9%)	81 (17.8%)	0.63 (0.34–1.18)		0.58 (0.29–1.17)	
	Dominant	A/A	44 (38.6%)	133 (29.2%)	1	0.055	1	0,15
		A/G-G/G	70 (61.4%)	323 (70.8%)	0.66 (0.43–1.00)		0.70 (0.44–1.13)	
	Recessive	A/A-A/G	97 (85.1%)	375 (82.2%)	1	0.46	1	0,24
		G/G	17 (14.9%)	81 (17.8%)	0.81 (0.46–1.43)		0.69 (0.36–1.30)	
	Over-dominant	A/A-G/G	61 (53.5%)	214 (46.9%)	1	0.21	1	0,64
		A/G	53 (46.5%)	242 (53.1%)	0.77 (0.51–1.16)		0.89 (0.57–1.42)	
	Log-additive	---	---	---	0.76 (0.56–1.04)	0.083	0.76 (0.54–1.06)	0,1

SNP, single nucleotide polymorphism; OR, odds ratio; 95% CI, 95% confidence interval.

* Values are calculated without adjustment dates.

** Values are calculated with adjusted for age and gender

*** *p-value < 0*.*05*, statistically significant

### Association between haplotypes and susceptibility to leprosy

**Fig 1:**
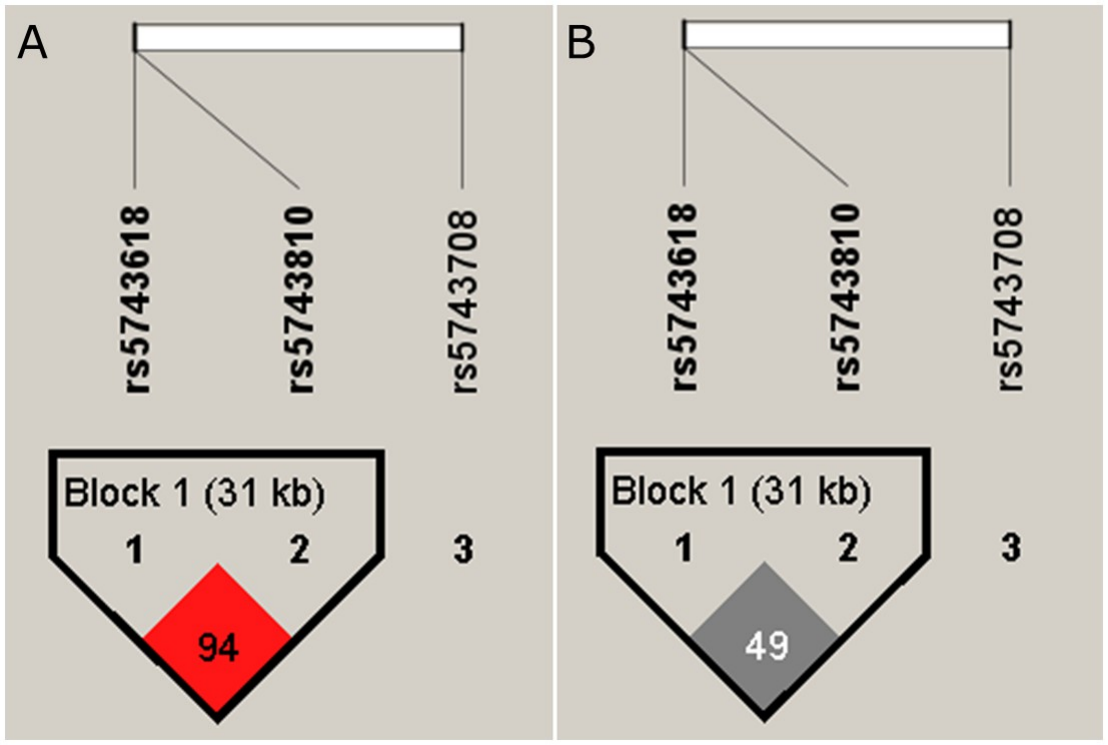
Haplotype block formed by the NOD2 gene. rs2111234, rs8057341, and rs3135499 SNPs. **A.** rs2111234 and rs8057341 SNPs form a block exhibiting moderate LOD (D´ = 89) **B.** Shows the same block with r2, values between rs2111234 and rs8057341 SNPs present an r2 = 0.79. The analysis was realized with Haploview v4.2.

Haplotypes with a greater than 1% frequency were included. The rs2111234 and rs3135499 SNPs presented a low LOD (linkage disequilibrium) with a D´ = 0.68 (*r*^*2*^ = 0.20) value, while rs2111234 and rs8057341 SNPs presented a moderate LOD with a D´ = 0.89 (*r*^*2*^ = 0.79) value**,** Fig 1. The GAG haplotype (rs2111234-G, rs3135499-A, rs8057341-G), with a frequency of 2.01%, represents a susceptibility factor for the development of leprosy (OR 3.17, IC95% 1.26–7.94; *p = 0*.*014*) in age and gender-adjusted data and (OR 3.30; IC95% 1.42–7.68, *p = 0*,*0059*) in not adjusted data, Table 4. Subgroup analyses showed that the same haplotype represents a susceptibility factor (OR 6.14, IC95% 1.59–23.7, *p = 0*.*0091*) in females (Table 5), but not in males S4 Table.

**Table 4:** Haplotype frequencies and association between rs2111234, rs135499, rs8057341 SNPs and susceptibility to leprosy (n = 570, adjusted for age and sex).

*SNP/Haplotype*	*rs2111234*	*rs3135499*	*rs8057341*	*Frequency*	*Not adjusted*		*Adjusted*	
					*OR (95% CI)*	*p-value* *	*OR (95% CI)*	*p*.-value**
*1*	*G*	*A*	*A*	*0*.*3618*	*1*	*---*	*1*	*---*
*2*	*A*	*C*	*G*	*0*.*3088*	*1*.*40 (0*.*94–2*.*07)*	*0*.*098*	*1*.*35 (0*.*88–2*.*08)*	*0*.*17*
*3*	*A*	*A*	*G*	*0*.*2352*	*1*.*47 (0*.*98–2*.*19)*	*0*.*062*	*1*.*31 (0*.*84–2*.*05)*	*0*.*24*
*4*	*G*	*C*	*A*	*0*.*0419*	*0*.*49 (0*.*14–1*.*73)*	*0*.*27*	*0*.*34 (0*.*09–1*.*25)*	*0*.*11*
*5*	*A*	*A*	*A*	*0*.*0215*	*1*.*63 (0*.*67–3*.*98)*	*0*.*28*	*2*.*34 (0*.*88–6*.*23)*	*0*.*089*
*6*	*G*	*A*	*G*	*0*.*0201*	***3*.*30 (1*.*42–7*.*68)*** ***	***0*.*0059***	***3*.*17 (1*.*26–7*.*94)*** ***	***0*.*014***
***Global haplotype association***					***p-value*: *0*.*061***		***p-value*: *0*.*0089***	

SNP, single nucleotide polymorphism; OR, odds ratio; 95% CI, 95% confidence interval.

* Values are calculated without adjustment dates.

** Values are calculated with adjusted for age and gender

*** p-value < 0.05, statistically significant

**Table 5:** Haplotypes association of the rs2111234, rs3135499, rs8057341 SNPs and susceptibility to leprosy in females (n = 283, adjusted for age).

Haplotype	rs2111234	rs3135499	rs8057341	Frequency	Not adjusted		Adjusted	
					OR (95% CI)	p-value*	OR (95% CI)	*p*.-value**
1	G	A	A	0.3634	1	---	1	---
2	A	C	G	0.3099	1.53 (0.85–2.77)	0.16	1.48 (0.80–2.76)	0.21
3	A	A	G	0.2298	1.65 (0.91–3.00)	0.1	1.50 (0.79–2.85)	0.21
4	G	C	A	0.0367	1.01 (0.22–4.67)	0.99	0.73 (0.15–3.63)	0.7
5	A	A	A	0.0292	2.32 (0.86–6.26)	0.097	2.69 (0.93–7.75)	0.068
6	G	A	G	0.0225	**6.75 (1.95–23.35)**	0.0028***	**6.14 (1.59–23.79)**	*0*.0091***
**Global haplotype association**					***p-value*:*0*.*015***		***p-value*:*0*.*041***	

### Functional analysis of SNPs

The GTEx database was used to analyze the functional effects of rs2111234, rs8057341, and rs3135499 SNPs in peripheral blood. The rs2111234 and rs8057341 variants affect NOD2 expression in peripheral blood, the possible functional effects of which were identified in the Regulome DB (https://www.regulomedb.org/). The rs3135499 and rs8057341 variants likely affect eQTL (expression quantitative trait loci) and transcription factor (TF)/ DNase peak binding. These were classified with a Regulome DB score of 1f, indicating that they are likely to affect binding and are linked in the expression of a target gene. The rs2111234 variant had a Regulome DB score of 2b, indicating that it probably affects binding, while the rs7194886 variant had a score of 4, which shows no evidence of affecting binding (Table 6).

**Table 6:** Functional rs3135499, rs2111234, rs8057341, and rs7194886 SNPs’ annotation results.

SNP	NES	Tissue	p-value	Regulome DB Score*	Function [25]
rs3199	-0.18	Whole blood	2.1 X10^-13	1f	Likely to affect binding and linked to expression of a gene target
	-0.23	Tibial nerve	2.5 X10^-8		
	-0.28	Skin—not sun-exposed (Suprapubic)	1.1X10^-23		
	-0.40	Esophagus–Mucosa	7.1X10^-35		
rs2111234	-0.52	Whole blood	2.4 x 10^-56	2b	Likely to affect binding
	-0.29	Tibial nerve	2.2 X10^-10		
rs8057341	-0.49	Whole blood	1x 10^-46	1f	Likely to affect binding and linked to expression of a gene target
	-0.29	Tibial nerve	3.1 X10^-10		
rs7194886	-0.25	Whole blood	2.1 x 10^-13	4	Minimal binding evidence
	-0.23	Tibial nerve	9.4 X10^-8		
	-0.28	Skin—not sun-exposed (Suprapubic)	9.8X10^-23		
	-0.35	Esophagus–Mucosa	9.0X10^-26		

* Scores were obtained from Regulome DB (http://www.regulomedb.org). 1f, eQTL + TF binding + matched TF motif + matched DNase Footprint + DNase peak; 2b, TF binding + any motif + DNase Footprint + DNase peak; 4, TF binding + DNase peak

## Discussion

*NOD2* is a pattern recognition receptor (PRR) that plays a key role in the presence and magnitude of the immune and inflammatory responses, thus determining infection outcome [22]. *NOD2* gene polymorphisms have been associated with susceptibility to leprosy and other immune-related diseases [21, 26, 27]. In this context, we performed a case-control study to evaluate the allele frequency and association of four polymorphisms (rs3135499, rs2111234, rs8057341, and rs7194886) in the *NOD2* gene and their relationship with susceptibility to leprosy in people born in the Norte de Santander region of Colombia, which is endemic to this disease [28]. This study shows that one of the analyzed SNPs, rs8057341, was associated with resistance to leprosy in a codominant model: A/A (OR 0.45; 95% CI 0.22–0.91, *p* = 0.044), dominant model: A/G-A/A (OR = 0.57; 95% CI = 0.36–0.92, *p = 0*.*021*), and additive model (OR 0.66; 95% CI = 0.47–0.0.92, *p* = 0.013). This finding indicates that the presence of a single A allele in this population confers resistance to developing this disease. When analyzed in other populations, this allele has been characterized as both a susceptibility and resistance factor.

A GWAS study on a Chinese population reported genetic variants associated with leprosy in different genes, which have been replicated in other populations [15]. In family-based and case-control studies in leprosy-endemic regions of Brazil, analyzed several of the markers found in the GWAS study associated with leprosy in the Chinese population. The rs8057341-A, rs2111234-G and rs3135499-C alleles were found to be associated with protection against leprosy in the study families from a leprosy-endemic colony in the state of Pará, northern Brazil [18]. The analysis in other Brazilian populations identified the AA genotype of the rs8057341 variant as a protective factor for leprosy development, Rondonópolis (OR 0.57; 95% CI 0.34–0.96, *p* = 0.03), Bauru (OR 0.37; 95% CI 0.21–0.64, *p* = 0.0003) and Río de Janeiro (OR 0.44; 95% CI 0.28–0.70, *p* = 0.004). A study analyzing individuals from Manaus, Brazil also found that the AA genotype (OR 0.56 95% CI 0.7–0.84, *p = 0*.*0052*) is a protective factor when data are adjusted for age, gender and ancestry [21].

Likewise, this SNP (rs8057341) showed association with the development of leprosy in a study conducted in a Vietnamese population [16]. In contrast, this variant was shown to be a risk factor (OR:1.17, 95% CI 1.09–1.26, *p = 5*.*53 x10*^*-5*^) in the Chinese study population [15]. These results show differences in susceptibility to leprosy in subjects carrying the rs8057341-A allele when different populations are analyzed, indicating the need to study each population and determine the role played by these SNPs in the development of the disease. Colombia is characterized by the great genetic diversity of its population, owing to the extent of past immigration [29, 30]. This study analyzed a population living in an endemic area of Colombia featuring 6.9% African ancestry, 58% European ancestry, and 34% native American ancestry [19].

On the other hand, the same association study performed in a Chinese population found that the rs7194886 SNP (OR 1.63, 95% CI 1.50–1.77, *p = 1*.*77 × 10*^*−30*^) and the rs3135499 SNP (OR 1.16, 95% CI 1.07–1.25, *p = 2*.*52 × 10*^*−4*^) related to leprosy susceptibility in that population [15]. In the present study, the rs7194886 allele was not in Hardy-Weinberg equilibrium in the control group (*p = 0*.*00088***),** which hindered the analysis. These two variants were also analyzed among the Chinese Yi population showing various associations [31]. The rs7194886 SNP showed no association (OR 1.23, 95% CI 0.91–1.68, *p = 0*.*178*), while the rs3135499 SNP (OR 2.55, 95% CI 1.83–3.55, *p = 1x10*^*-8*^) showed higher significance [31]. While in the present study, analyzing a population in Norte de Santander, the rs3135499 variant was not shown to be associated with the development of leprosy; these results together show that the rs8057341-A variant of the NOD2 gene is a resistance factor for leprosy development in the Norte de Santander population.

When performing an analysis of the three associated SNPs among the study population, it was found that the GAG (-234G, -499A, -341G) haplotype behaves as a susceptibility factor for the development of the disease, especially when adjusted for sex and age (Table 4). Additionally, in the subgroup analysis, the GAG haplotype (-234G, -499A, -341G) was of higher risk in women (Table 5). In this study, the proportion of female cases (49%) was similar to that of male cases (51%); indicating that this was not the reason underlying the higher risk associated with this haplotype in women. In Colombia, the proportion of males diagnosed with leprosy in 2021 was 60.94 [20]; therefore, we suggest that an explanation for this finding is the higher proportion of men carrying the GAG haplotype in this Colombian population, which is difficult to verify due to the high total population in Colombia and the fact that our study population was selected based on convenience.

*In silico* analyses show that the four SNPs decrease protein expression levels in different tissues. The rs3135499 variant located in the 3´UTR region and the rs8057341 variant located in the intron region (Haploreg v4.1) may affect the secondary structure of these regions and dysregulate NOD2 expression [32]. Thus, a decrease in expression of 18% to 28% for the rs3135499 SNP and 29% to 49% for the rs8057341 SNP was observed and affects the binding site (Table 6). In monocytes, the NOD2 receptor is involved in the recognition and activation of the mycobacterial muramyl dipeptide, which induces IL-32 expression, thereby regulating monocytes’ CD1b+ dendritic cell expression. However, the functional properties of the NOD2 gene variants herein described and their relationship to the pathophysiology of leprosy remain unknown [33].

The NOD2 protein also participates in autophagy. NOD2 activation induces the formation of an autophagosome, mediated by r ATG16L1 protein complex recruitment [34] to the plasma membrane, which initiates autophagy in the host cells [35]. NOD1- and NOD2-deficient-mice demonstrate increased susceptibility to various pathogens [36, 37]. Since this protein is involved in NF-κB and MAPK signaling pathway activation [38, 39]. Likewise, NOPD2 regulates Th17 cell responses [40]. NOD2-mediated antigenic recognition leads to Th2-dependent adaptive immune responses [41] These NOD2 gene functions show that changes in gene expression, as shown in the in silico analyses of the rs3135499 and rs8057341 variants, may play a role in the host immune response. Furthermore, since this protein participates in the activation of various signaling pathways, its impact on the susceptibility to immune disease depends on its interactions with other proteins and the genetic background of the studied population.

This is the first study of a leprosy-endemic region in Columbia to determine which *NOD2* gene polymorphisms predispose individuals to leprosy and which protect against it, and to compare these tendencies to those obtained in other parts of the world. Any observed differences in these results could be related to differences in race and geographic location.

The present study has some limitations. It was conducted in a single population in the northern region of Colombia and, therefore, does not represent all of the leprosy-endemic regions across Colombia, particularly given the great genetic diversity harbored by the Colombian population. Moreover, since we focused on genetic analyses of SNPs and their association with leprosy, the mechanisms through which the associated SNPs generate resistance or susceptibility to leprosy remain unclear. Overall, these findings further our understanding of population-based genetic factors that play a role in leprosy susceptibility. Future studies in other leprosy-endemic regions of Colombia are warranted.

## Supporting information

S1 Table STrengthening the REporting of Genetic Association studies (STREGA) reporting recommendations, extended from STROBE Statement. (DOCX)

S2 Table Data collection form. (PDF)

S3 Table Sequence of primers used in the study to amplification segments for analysis of each SNP. (DOCX)

S4 Table Association of haplotypes of the SNPs rs2111234, rs3135499, rs8057341 and susceptibility to leprosy in males. (DOCX)
