# Supplementary material for: Association of the rs2111234, rs3135499, rs8057341 polymorphisms in the NOD2 gene with leprosy: A case-control study in the Norte de Santander, Colombia population
Source: PLoS One. 2023 Mar 6;18(3):e0281553. doi: 10.1371/journal.pone.0281553 (PMC9987820; doi:10.1371/journal.pone.0281553)
Supplement: S2 Table — (PDF) [file pone.0281553.s002.pdf]

DETERMINACIÓN DE POLIMORFISMOS GENÉTICOS ASOCIADOS CON SUSCEPTIBILIDAD PARA ENFERMEDAD DE HANSEN EN POBLACIÓN DEL DEPARTAMENTO DE NORTE DE SANTANDER.

**codigo ID**	
**NOMBRE**	
**APELLIDO**	
**APELLIDO2**	
**ID**	
**identificacion**	
**fecha notificacion**	
**registro EN EL ESTUDIO**	
**Municipio de residencia**	
**direccion contacto**	
**telefono contacto**	
**Estado caso o control**	
**Edad años**	
**sexo**	
**Municipio de nacimiento**	
**Municipio de procedencia**	
**Municipio de nacimiento del padre**	
**Municipio de nacimiento de la madre**	
**Clasificación de la enfermedad en casos**	
**Etnia**	
**rs3088362-Adenina**	
**rs3088362-Timina**	
**rs3088362-Guanina**	
**rs3088362-Citocina**	
**rs3088362-Adenina homocigoto**	
**rs3088362-Timina homocigoto**	
**rs3088362-Guanina homocigoto**	
**rs3088362-Citocina homocigoto**	
**rs3764147-Adenina**	
**rs3764147-Timina**	
**rs3764147-Guanina**	
**rs3764147-Citocina**	
**rs3764147-Adenina homocigoto**	
**rs3764147-Timina homocigoto**	
**rs3764147-Guanina homocigoto**	
**rs3764147-Citocina homocigoto**	
**rs10507522-Adenina**	
**rs10507522-Timina**	
**rs10507522-Guanina**	
**rs10507522-Citocina**	
**rs10507522-Adenina homocigoto**	
**rs10507522-Timina homocigoto**	
**rs10507522-Guanina homocigoto**	
**rs10507522-Citocina homocigoto**	

DETERMINACIÓN DE POLIMORFISMOS GENÉTICOS ASOCIADOS CON SUSCEPTIBILIDAD PARA ENFERMEDAD DE HANSEN EN POBLACIÓN DEL DEPARTAMENTO DE NORTE DE SANTANDER.

**rs9302752-Adenina**	
**rs9302752-Timina**	
**rs9302752-Guanina**	
**rs9302752-Citocina**	
**rs9302752-Adenina homocigoto**	
**rs9302752-Timina homocigoto**	
**rs9302752-Guanina homocigoto**	
**rs9302752-Citocina homocigoto**	
**rs7194886-Adenina**	
**rs7194886-Timina**	
**rs7194886-Guanina**	
**rs7194886-Citocina**	
**rs7194886-Adenina homocigoto**	
**rs7194886-Timina homocigoto**	
**rs7194886-Guanina homocigoto**	
**rs7194886-Citocina homocigoto**	
**rs4574921-Adenina**	
**rs4574921-Timina**	
**rs4574921-Guanina**	
**rs4574921-Citocina**	
**rs4574921-Adenina homocigoto**	
**rs4574921-Timina homocigoto**	
**rs4574921-Guanina homocigoto**	
**rs4574921-Citocina homocigoto**	
**rs10114470-Adenina**	
**rs10114470-Timina**	
**rs10114470-Guanina**	
**rs10114470-Citocina**	
**rs10114470-Adenina homocigoto**	
**rs10114470-Timina homocigoto**	
**rs10114470-Guanina homocigoto**	
**rs10114470-Citocina homocigoto**	
**rs6478108-Adenina**	
**rs6478108-Timina**	
**rs6478108-Guanina**	
**rs6478108-Citocina**	
**rs6478108-Adenina homocigoto**	
**rs6478108-Timina homocigoto**	
**rs6478108-Guanina homocigoto**	
**rs6478108-Citocina homocigoto**	
