# Supplementary material for: Association of the rs2111234, rs3135499, rs8057341 polymorphisms in the NOD2 gene with leprosy: A case-control study in the Norte de Santander, Colombia population
Source: PLoS One. 2023 Mar 6;18(3):e0281553. doi: 10.1371/journal.pone.0281553 (PMC9987820; doi:10.1371/journal.pone.0281553)
Supplement: S3 Table — (DOCX) [file pone.0281553.s003.docx]

**Table S3** Sequence of primers used in the study to amplification segments for analysis of each SNP

| **SNP** | **Forward**  **(5´- 3´) DNA** | **Reverse**  **(5´- 3´) DNA** | **Size (pb)** |
| --- | --- | --- | --- |
| NOD2 (rs7194886) | 5´CCAGAGGCCAAACAG CAATT-3´ | CATCTTCTC TCAGCC CCACT | 346bp |
| NOD2 (rs2111234) | GTC GAG AAC ATG CTG GAC CTG | TTG AGC GCA GGA ATT CAA GG | 371bp |
| NOD2(rs8057341) | AGG GAC TTG GTC TGG CTT TT | AGATGGCCACCA GTT CAA ACA | 447pb |
| NOD2 (rs3135499) | GGC AGC CTC TTC AAA ATG AG | GCA GGC TAA GGT GTA GCC CTT C | 558pb |
