# Supplementary material for: Association of the rs2111234, rs3135499, rs8057341 polymorphisms in the NOD2 gene with leprosy: A case-control study in the Norte de Santander, Colombia population
Source: PLoS One. 2023 Mar 6;18(3):e0281553. doi: 10.1371/journal.pone.0281553 (PMC9987820; doi:10.1371/journal.pone.0281553)
Supplement: S4 Table — (DOCX) [file pone.0281553.s004.docx]

**Table** **S4** Association of haplotypes of the SNPs rs2111234, rs3135499, rs8057341 and susceptibility to leprosy in males (n=283, adjusted for age)

| **SNP/Haplotype** | **SNP rs2111234** | **rs3135499** | **rs8057341** | **Frequency** | **OR (95% CI)** | ***p-value*** |
| --- | --- | --- | --- | --- | --- | --- |
| 1 | G | A | A | 0.3633 | 1 | --- |
| 2 | A | C | G | 0.3086 | 1.25 (0.66 - 2.33) | 0.49 |
| 3 | A | A | G | 0.2397 | 1.20 (0.63 - 2.27) | 0.58 |
| 4 | G | C | A | 0.0439 | 0.20 (0.02 - 1.67) | 0.14 |
| 5 | G | A | G | 0.0182 | 1.49 (0.31 - 7.11) | 0.62 |
| 6 | A | A | A | 0.0112 | 1.28 (0.05 - 34.83) | 0.88 |
| 7 | A | C | A | 0.0102 | 0.78 (0.01 - 119.41) | 0.92 |
| ***Global haplotype association p-value: 0.3*** | | | | | | |
